# Contributions to medical literature by Indus Hospital Health Network through Research

**DOI:** 10.12669/pjms.38.ICON-2022.5757

**Published:** 2022-01

**Authors:** Shaukat Ali Jawaid

Ever since the establishment of Indus Hospital Health Network (IHHN) by Dr. Abdul Bari and his colleagues, they have made a commendable progress and practically demonstrated that provision of free tertiary care is possible with the help of philanthropists and donors. It only becomes possible once an institution has earned credibility and the donors are confident that their contributions are being used judiciously for the purpose they are made.

Starting with a major hospital at Karachi, IHHN has now expanded its network in the country and this institution has emerged as an important non-governmental player in health sector. It offers treatment facilities in almost every discipline of medicine with a multidisciplinary team approach with exceptional results. Apart from providing healthcare, it also realized the importance of documentation and publication for which they established a Research Department. Hospitals in Pakistan are considered as goldmine of data offering innumerable opportunities for research but most of them lack proper record keeping hence the data needed for research publications is not easy to find. However, IHHN knowing its importance managed to install the Health Information Management System which makes their researcher’s job easy. Since the researchers are also lucky to get help, guidance and assistance from the Research Department, IHHN faculty members and postgraduate trainees have managed to complete numerous research project thus showcasing their research work at international conferences which IHHN holds regularly and much of this research work is also published.

We in Pakistan Journal of Medical Sciences published a special issue at the time of their last conference ICON 2021. This year too, we were approached and after initial discussions on working modalities, the project was started in March 2021.As agreed IHHN provided us manuscript after peer review by two reviewers which were then finally reviewed and edited by our editorial team before they were accepted for publication in this “Only Online Edition”. The workshop that we organized at Indus Hospital on Medical Writing and Peer Review during 2020 was very helpful and the experience which the IHHN Research Department gained while working with us last year proved very helpful with the result that this time it was mostly a smooth sailing.

This special issue covers some very important subjects. Faiza Ahmed and colleagues in their write up has highlighted the knowledge and perceptions of sepsis among doctors. Sepsis, they have concluded remains a challenge to treat, it is often misdiagnosed. Early diagnosis and prompt management is the key to improving the outcome. 1

Asthma severity and hospital admissions measured by PRAM score have been discussed by Kazi et al. Their conclusions are that this score is the best predictor for the need for hospitalization for these patients suffering from asthma. They have suggested that this should be put to use in clinical practice in paediatric emergency departments. They suggest better facilitation of intensive therapy of patients at triage which will decrease the need for hospitalization.^2^

Obstructive lung disease is an important NCD. According to Saima Saeed and colleagues the OLD programme at IHHN uses capacity building, gold standard diagnostic and management strategies in primary care. This enables early diagnosis of suspected patients which improves the morbidity and mortality. 3

Drug resistant tuberculosis remains yet another important problem in Paksitan. Nazia Khursheed and colleagues have identified in their study a number of such cases. They have advocated development of new strategies to reduce the spread of this disease.^4^

Safety and efficacy of Remdesivir on mortality and length of stay in hospitalized Covid19 patients has been studied by Quratulain Shaikh et al., in this prospective cohort study which enrolled two hundred sixty eight patients. The findings of this study showed that there was no difference in in-hospital mortality while length of stay was longer in these patents. In addition more patients had severe ARDS in the RDV group. 5

Ensuring privacy and confidentiality while examining a patient is extremely important but unfortunately practically it is not possible in overcrowded Out Patients Departments of public hospitals. Syed Ghuzzanfar Saleem et al studied the patient’s perceptions on this issue who visited the emergency department of Indus Hospital Adult Emergency Department. They found out that despite overcrowded and busy environment patients generally felt that privacy and confidentiality were maintained which must be very satisfying for the hospital administration.^6^

There are numerous other studies covered in this special issue which shed light on many important subjects which the researchers have discussed which make an important reading. We are delighted to be a part of this project and by offering highly concessional publication charges despite the massive devaluation of the currency, we have contributed our share to promote and project the image of this remarkable institution of which the country should be proud of. In future too, we will be delighted to help, assist and cooperate with IHHN to showcase its research work thereby making significant contribution to the world medical literature from Pakistan.

We are extremely grateful to the management of IHHN for providing us this opportunity and the two Guest Editors Dr. Farhana Amanullah and Dr. Naila Baig Ansari for their hard work in compiling, this special supplement.

## References

[1] Ahmed F, Abbasi L, Herekar F, Jiwani A, Patel MJ (2022). Knowledge and perception of Sepsis among Doctors in Karachi Pakistan. Pak J Med Sci.

[2] Kazi U, Gul Rukh S, Zawawi S, Laila S, Fareeduddin M, Saleem SG (2022). To determine the association between asthma severity and hospital admission measured by Pediatric Respiratory Assessment Measure (PRAM) score at Indus Hospital and Health Network, Karachi, Pakistan, 2020-2021. Pak J Med Sci.

[3] Saeed S, Siddiqui M, Altaf R (2022). The Obstructive Lung Diseases Program:Integrated obstructive lung disease services within primary care in Pakistan. Pak J Med Sci.

[4] Khursheed N, Asif S, Bano S, Ali MM, Adnan F (2022). Susceptibility pattern of Mycobacterium tuberculosis over a period of five years at Indus Hospital and Health Network, Karachi, Pakistan. Pak J Med Sci.

[5] Shaikh Q, Sarfaraz S, Rahim A, Hussain M, Shah R, Soomro S (2022). Effect of Remdesivir on mortality and length of stay in hospitalized COVID-19 patients:A single center study. Pak J Med Sci.

[6] Saleem SG, Ali S, Ghouri N, Maroof Q, Jamal MI, Aziz T (2022). Patient perception regarding privacy and confidentiality:A study from the emergency department of a tertiary care hospital in Karachi, Pakistan. Pak J Med Sci.

